# Precipitation, vegetation productivity, and human impacts control home range size of elephants in dryland systems in northern Namibia

**DOI:** 10.1002/ece3.9288

**Published:** 2022-09-13

**Authors:** Lorena Benitez, J. Werner Kilian, George Wittemyer, Lacey F. Hughey, Chris H. Fleming, Peter Leimgruber, Pierre du Preez, Jared A. Stabach

**Affiliations:** ^1^ Smithsonian National Zoo & Conservation Biology Institute Front Royal Virginia USA; ^2^ Etosha Ecological Institute, Ministry of Environment, Forestry and Tourism Okaukuejo Namibia; ^3^ Department of Fish, Wildlife, and Conservation Biology Colorado State University Fort Collins Colorado USA; ^4^ Save the Elephants Nairobi Kenya; ^5^ Department of Biology University of Maryland Maryland USA; ^6^ African Wildlife Conservation Trust Windhoek Namibia

**Keywords:** elephants, home range, movement, Namibia

## Abstract

Climatic variability, resource availability, and anthropogenic impacts heavily influence an animal's home range. This makes home range size an effective metric for understanding how variation in environmental factors alter the behavior and spatial distribution of animals. In this study, we estimated home range size of African elephants (*Loxodonta africana*) across four sites in Namibia, along a gradient of precipitation and human impact, and investigated how these gradients influence the home range size on regional and site scales. Additionally, we estimated the time individuals spent within protected area boundaries. The mean 50% autocorrelated kernel density estimate for home range was 2200 km^2^ [95% CI:1500–3100 km^2^]. Regionally, precipitation and vegetation were the strongest predictors of home range size, accounting for a combined 53% of observed variation. However, different environmental covariates explained home range variation at each site. Precipitation predicted most variation (up to 74%) in home range sizes (*n* = 66) in the drier western sites, while human impacts explained 71% of the variation in home range sizes (*n* = 10) in Namibia's portion of the Kavango‐Zambezi Transfrontier Conservation Area. Elephants in all study areas maintained high fidelity to protected areas, spending an average of 85% of time tracked on protected lands. These results suggest that while most elephant space use in Namibia is driven by natural dynamics, some elephants are experiencing changes in space use due to human modification.

## Graphical abstract

We analyze the largest dataset of collared elephants ever recorded in Namibia to determine the relative importance of humans and environmental variables in shaping elephant movements. We found that human activities are influencing elephant space use at some sites, though regional trends are still driven by precipitation and vegetation productivity.

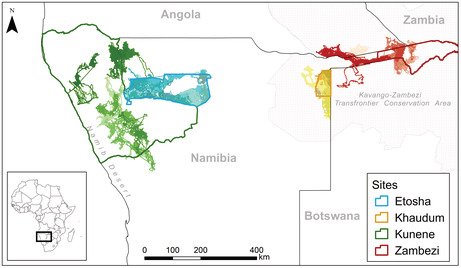

## 1 INTRODUCTION

Home range is a fundamental concept of ecology, used to characterize space use patterns of animals and has been defined as the total area required to meet nutritional and reproductive needs throughout an animal's lifetime (Burt, 1943). Home‐range estimation is a potentially useful metric for defining the appropriate size of protected areas or for understanding how environmental factors impact the behavior of individuals and the spatial distribution of populations (Börger et al., 2006). However, home range size varies immensely across species, populations, and individuals. While interspecific variation is primarily attributed to differing metabolic requirements (Carbone et al., 2005; Harestad & Bunnell, 1979; Kelt & van Vuren, 2001; Noonan et al., 2020), intraspecific variation is far less understood (Seigle‐Ferrand et al., 2021).

A variety of factors have been shown to best characterize variation in home range size amongst individuals of the same species (Börger et al., 2008; McLoughlin & Ferguson, 2000). Variation at the population and individual level has been linked to intrinsic (e.g., age, sex, number of offspring, conspecifics, personality) and extrinsic (e.g., resource availability, climate, terrain) factors (Kie et al., 2002; Morellet et al., 2013; Rivrud et al., 2010; Schirmer et al., 2019; van Beest et al., 2011; Wall et al., 2021; Wittemyer et al., 2008). However, there is a lack of studies which examine how ecological drivers contribute to individual variation in home range size within a region (Seigle‐Ferrand et al., 2021). Understanding how these factors influence home range size and space use is important for informing the management and conservation of threatened species. This is especially true for large‐bodied mammals, such as elephants (*Loxodonta africana*), which move long distances and are more susceptible to extinction as a result (Cardillo et al., 2005).

African savannah elephants are the world's largest terrestrial animal and are a species of high conservation concern (Thouless et al., 2016). Once widespread across the African continent, elephants are now largely restricted to isolated protected areas, with their distribution limited mostly by human encroachment rather than environmental conditions (Wall et al., 2021). This restriction of their range, along with poaching, has led to a steep decline in elephant numbers across Africa ‐ a reduction to approximately 118,000 elephants in 10 years (2007–2016; Thouless et al., 2016). The species was recently downgraded from Vulnerable to Endangered on the IUCN Red List (Gobush et al., 2021), with continental population estimates declining by more than 50% in the past two generations (50 years; Gobush et al., 2021). Elephants are important ecosystem engineers, found across a variety of habitats from deserts to tropical forests (Haynes, 2012). Because they inhabit many ecosystems, the size of elephant home ranges can differ dramatically between populations. For example, elephants in the deserts of Mali can have home ranges up to 32,000 km^2^ (Wall et al., 2013), while the largest home ranges in the wet savannahs of Uganda are closer to 500 km^2^ (Grogan et al., 2020). The disparity between populations highlights the need for population‐level studies to assess the relationship between home range size and environmental factors to better understand factors driving differences in spatial requirements. It is especially important to compare populations at multiple scales and across gradients of land use and ecological conditions to best understand the scale at which elephants are influenced by anthropogenic and environmental factors.

In this study, we examined home range size of elephants in northern Namibia, an important stronghold for the species where elephant numbers have more than doubled since 1995 (Thouless et al., 2016). Elephants span a diverse mosaic of land uses and environmental conditions across Namibia, which consists of a vast network of protected areas that include both multiuse communal conservancies and formally protected national parks. Elephants within Namibia are found from the hyper‐arid ecosystems in the west to the flooded grassland, savannah, and woodland habitats in the east. We expand upon past research by analyzing the largest dataset ever recorded (*n* = 86) on the movements of elephants across Namibian ecosystems. We tested the hypothesis that elephant home ranges correspond to extrinsic environmental factors which vary geographically across Namibia as found in other parts of Africa (Loarie et al., 2009). We focused on home range size as a core ecological process to assess the space use needs of each population, with inference on the environmental factors that influence variability. While in some ways similar to a resource selection and/or step‐selection function analysis (Boyce & McDonald, 1999; Manly et al., 2002; Roever et al., 2012; Thurfjell et al., 2014; Van Moorter et al., 2016), our analysis does not examine individual decisions (the points and turning angles) that animals make and builds upon previous work conducted across regional scales to assess elephant space use (Buchholtz et al., 2019; de Beer & van Aarde, 2008; Roever et al., 2012; Young et al., 2009).

To test our hypothesis, we incorporated Global Positioning System (GPS) telemetry data collected from elephants in four populations between 2008 and 2015. We combined these data with environmental variables, measured from remotely sensed data, to assess how each variable impacts home range size at regional and site‐level scales. Specifically, we tested the relative influence of precipitation, surface water, vegetation, human impact, and the amount of area protected on the variation in elephant home range size. Because of the overwhelming importance of water resources in arid systems (Wall et al., 2013), we hypothesized that differences between populations would primarily be driven by precipitation, while site‐level variation would be best explained by the availability of forage resources and extent of anthropogenic footprint (Wall et al., 2021). By determining how natural and anthropogenic factors influence elephant space use at multiple scales, we aim to shed light on conservation successes and areas for concern for elephant management in Namibia.

## 2 MATERIALS AND METHODS

### 2.1 Study sites

**FIGURE 1:**
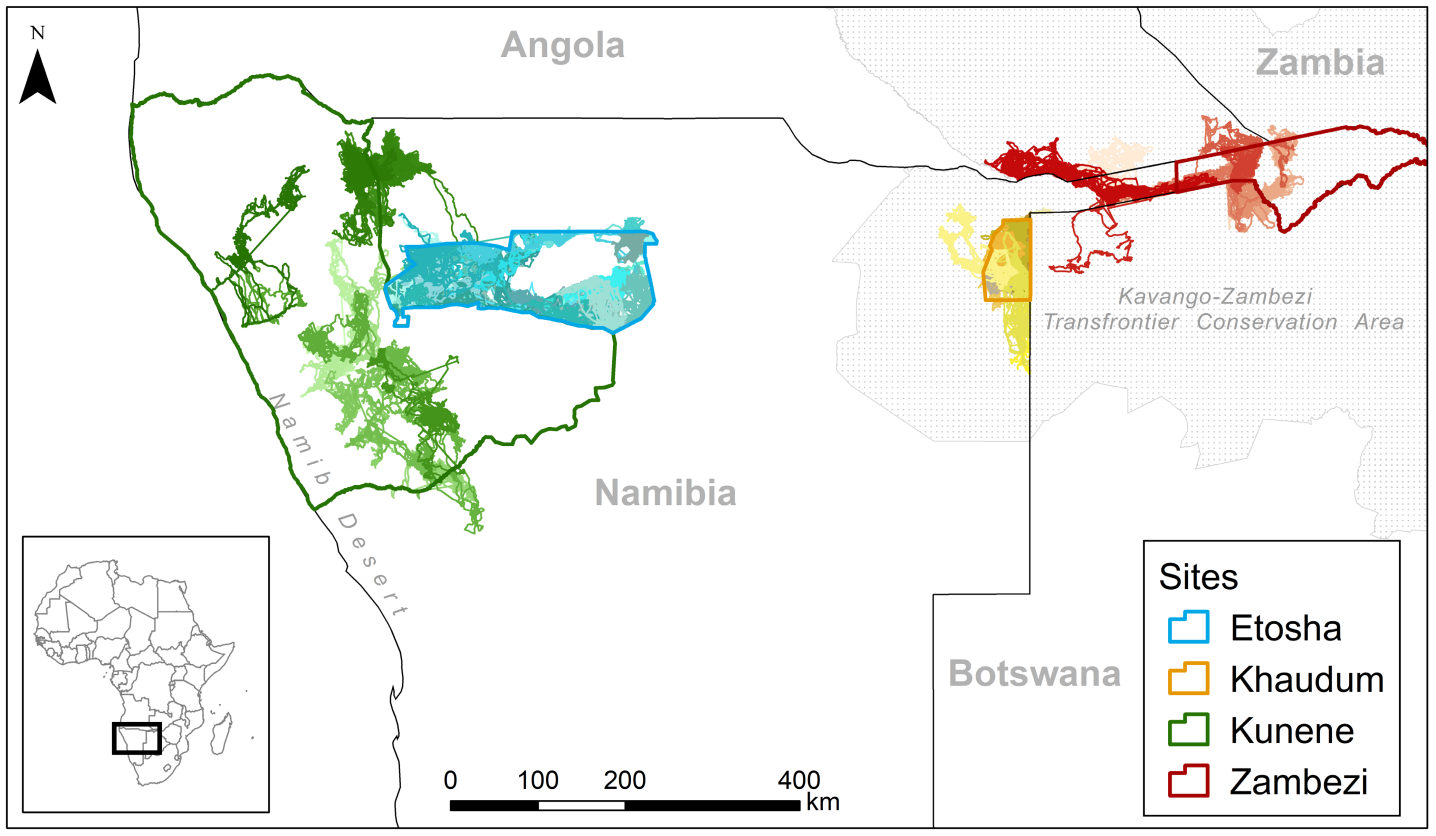
The maps of four local sites with total tracks of elephants indicated from Kunene (green), Etosha (aqua), Khaudum (yellow), and Zambezi (red)

This study compares data from four sites along an east–west gradient within the arid to semi‐arid savanna region of Southern Africa (25°15′45.3′′E to 11°44’10.3′′E). The furthest east, and by extension the wettest, site is the Zambezi region of Namibia (henceforth referred to as Zambezi; Figure 1). The site is part of the Kavango‐Zambezi Transfrontier Conservation Area (KAZA) and is composed of several national parks, conservancies, and forest reserves, some of which connect to adjacent protected areas in Botswana, Angola, and Zambia. The site receives 607 ± 59 mm of rainfall annually (Funk et al., 2015; Appendix S2: Figure A1). An estimated 12,000 elephants reside in this part of Namibia (Craig & Gibson, 2019a, 2019b). The site has the highest density of humans (6.2 people/km^2^; Namibia Statistics Agency, 2011) of our study areas and greatest human modification (Kennedy et al., 2019; Appendix S2: Figure A2).

Just west of Zambezi lies Khaudum National Park (henceforth Khaudum; Figure 1). The 3841 km^2^ protected area is directly adjacent to the fenced Namibia‐Botswana border and receives 540 ± 19 mm of rainfall annually (Funk et al., 2015; Appendix S2: Figure A1). Khaudum shares an open southern border with the Nyae Nyae community conservancy and is estimated to support approximately 8000 elephants (Craig & Gibson, 2019a, 2019b). The human population density in the Kavango region around Khaudum is approximately 4.6 people/km^2^ (Namibia Statistics Agency, 2011).

Further west, Etosha National Park is a 22,270 km^2^ protected area located in north‐central Namibia (henceforth Etosha; Figure 1). Etosha is a semi‐arid savannah with approximately 394 ± 52 mm of rainfall annually (Funk et al., 2015; Appendix S2: Figure A1). The Park has been fenced since the early 1970’s and is estimated to support an elephant population of approximately 2900 animals (Kilian, 2015). There are limited influences from humans within the park, but Etosha borders some regions with high human population densities (>20 people/km^2^), though most surrounding regions have low population density (<1 person/km^2^; Namibia Statistics Agency, 2011).

Our driest study site was the Kunene region of northwestern Namibia (henceforth referred to as Kunene; Figure 1). Kunene is arid with much of its area lying within the Namib and pro‐Namib desert. The site receives 209 ± 119 mm of rainfall annually (Funk et al., 2015; Appendix S2: Figure A1), consisting of a patchwork of multiuse conservancies and more restricted concessions that support approximately 1100 elephants (Craig & Gibson, 2016). It has the lowest human population density of our study areas (0.8 people/km^2^) due to its aridity and limited options for agriculture (Namibia Statistics Agency, 2011).

### 2.2 Elephant movement data

A total of 86 elephants were captured and fitted with a GPS‐satellite transmitter between 2008 and 2013 across the four study sites. Elephants were grouped by site based on where they were collared. Females were from different family groups. Male elephants were either single or from small groups consisting of males only. All capture and collaring procedures were performed by veterinarians from the Namibian Ministry of Environment and Tourism, following South African National Standards for animal welfare and care (SABS, 2000).

In Zambezi, nine cows and one bull were collared in 2010 with data collected until 2014. Location information was recorded at 60‐minute intervals. The average tracking period was 780 days (SD = 190) with 15,554 GPS location fixes collected. In Khaudum, 10 cows were collared in 2012 and 2013 with data collected until 2015. Location information was also recorded at 60‐min intervals. The average tracking period was 620 days (SD = 111) with 11,505 GPS fixes collected.

Within Etosha, 39 elephants (22 cows, 17 bulls) were fitted with GPS collars between 2008 and 2013, with data collected until 2015. Location information was recorded at 15‐, 20‐ or 30‐min intervals. The average tracking period was 726 days (SD = 310) with 62,722 GPS fixes collected. In Kunene, 27 elephants (15 cows, 12 bulls) were tracked for approximately 2 years beginning in December 2010. Location information was recorded at 30‐minute intervals. The average tracking period was 741 days (SD = 172). A total of 35,297 GPS fixes were collected.

The final dataset across all sites consisted of 3,669,784 GPS fixes spanning 8 years (2008–2015). For individual elephants, the number of GPS fixes ranged from 5090 to 209,942, with a median value of 36,809 points. The first day of the tracking period was removed from each dataset to eliminate unusual movement behavior caused by collaring procedures (Northrup et al., 2014). A summary of the tracking data is provided in the Appendices S1 and S2.

### 2.3 Environmental predictors

Landscape information for vegetation, precipitation, surface water availability, protected area designation, and human impact were collected from globally available data layers, and processed in Google Earth Engine (Gorelick et al., 2017). Data from multiple sources and indices were used for each criterion to test which method best quantifies differences between the four sites. Mean and standard deviation values for variables included in resulting models are provided in Table 1 for each site.

**TABLE 1:** The mean and standard deviations (in parentheses) of each environmental variable calculated using polygons derived by the combined 99% AKDE home ranges for individuals at each site

Site	Area (km^2^)	NDVI	Human modification	Annual Precipitation (mm)	Surface Water Occurrence	Protected Area (%)	National Park (%)
Kunene	122,000	0.192 (0.0839)	0.0619 (0.082)	212 (123)	24.3 (28.4)	84.8	16.4
Etosha	86,800	0.285 (0.0731)	0.0907 (0.105)	390 (101)	13.5 (9.53)	47.7	26.4
Khaudum	23,700	0.383 (0.0357)	0.0748 (0.0681)	539 (19.8)	4.78 (2.83)	58.2	16.2
Zambezi	65,800	0.418 (0.0500)	0.155 (0.155)	595 (60.4)	18.2 (28.0)	34.7	25.0

We tested three MODIS‐derived vegetation indices to quantify vegetation availability and variability: normalized difference vegetation index (NDVI), modified soil‐adjusted vegetation index (MSAVI), and fraction of photosynthetically active radiation (FPAR). NDVI measures green biomass and vegetation productivity (Pettorelli, 2013). Because NDVI is less reliable in arid and semiarid areas due to the effect of bare soil (Boschetti et al., 2007), MSAVI was included as an alternative. MSAVI increases the dynamic range of vegetation signals and reduces the influence of soil background to better estimate vegetation in arid habitats (Qi et al., 1994). FPAR is a measure of the proportion of sun radiation received by a plant to the total available photosynthetically active wavelengths of radiation (Knyazikhin, 1999). For arid areas like Namibia, FPAR is expected to be a better predictor of herbaceous biomass as it encompasses both green and dry biomass (Tsalyuk et al., 2015).

The impact of water was examined both in terms of rainfall and available surface water. Precipitation estimates were extracted from the Climate Hazards Group InfraRed Precipitation with Station (CHIRPS) dataset, which estimates daily rainfall at 0.05° resolution (Funk et al., 2015). Annual mean precipitation was calculated from 2008 to 2015 for the study area. Surface‐water availability was represented using the JRC Global Surface Water Mapping Layers (30‐m resolution), providing data on the location and temporal distribution of surface water from 1984 to 2019 (Pekel et al., 2016). Bands for occurrence (the frequency with which water was present) and seasonality (how many months water is present) were used as variables in our analysis. In addition, we calculated the location of permanent and seasonal water sources by filtering the seasonality layer to pixels where water presence is greater than (permanent) and less than (seasonal) 9 months of the year.

Two data layers were used to represent human impact at 1‐km resolution: Human Footprint (HF; Venter et al., 2016) and global Human Modification (HM; Kennedy et al., 2019). HF is an index of human pressures derived from the summation of eight data layers approximately representing human impact for 2009. HM is a metric for the proportion of a landscape that has been modified by humans and based on an existing threat classification system by Salafsky et al. (2008) for 2016. The layers differ significantly in how they are calculated (Oakleaf & Kennedy, 2018) and emphasize different aspects of human impacts (e.g., HF focuses more heavily on roads than HM). Data on road location and road type were included from the Global Roads Inventory Project (GRIP) dataset (Meijer et al., 2018). Protected area designations were derived from UNEP (UNEP‐WCMC, 2018).

### 2.4 Home range and movement

We calculated variograms, fit continuous‐time movement models, and estimated home‐range sizes using the *ctmm* package (Calabrese et al., 2016) in R (R Development Core Team, 2020). Due to the amount of data being analyzed, we fit all models using the Smithsonian Institution's High Performance Computing Cluster (Smithsonian Institution, 2020). We first plotted the estimated semivariance function for each individual to assess the autocorrelation structure of the data (Fleming et al., 2014a). Resulting semi‐variograms were visually inspected for each animal to determine whether the data reached an asymptote, indicating whether animals met the range residency assumption (Calabrese et al., 2016). The semivariance function's curvature at short time lags was used to indicate whether or not the data could support velocity estimation. Models were fit using residual maximum likelihood and ranked using AICc (Fleming et al., 2019). We estimated home ranges conditional on the best‐fit model for each individual using auto‐correlated kernel density estimation (AKDE) at the 50% coverage level (Fleming et al., 2014b, 2018; Fleming & Calabrese, 2017). Accounting for autocorrelation in home‐range estimation is especially important for elephants, given the recognized underestimation of species area requirements due to the animals' large body size (Noonan et al., 2020). For comparative purposes with historic range estimates, we also calculated minimum convex polygons for each elephant using the *adehabitatHR* package (Calenge, 2006).

### 2.5 Statistical analyses

To assess variation in home‐range sizes, we compared AKDE estimates at the 50% level using the *meta* function in *ctmm*, which estimates population‐level parameters from individual‐level parameter estimates while taking into account estimate uncertainty (Fleming et al., 2022). We use this method to compare home range size between sexes and sites. An analysis of variance (ANOVA) was used to determine whether there were significant differences between groups (*p* < .05).

To summarize the environmental data layers, we used zonal statistics (function e*xactextractr*, Baston, 2019) to calculate the mean and standard deviation within each individual's home‐range polygon. The total length of roads and rivers within each home range were estimated and divided by home‐range area to standardize estimates across individuals. Percentages of each protected area designation (national park, concession, communal conservancy, and forest reserve) within each home range were also calculated.

To determine which environmental variables drive variation in home‐range size across elephant populations, we used generalized linear models (GLM). We eliminated highly correlated variables within each environmental category (vegetation, precipitation, surface water, human impact, and protected area) by first conducting univariate regressions, ranking individual models based on AIC to determine the best variable within each category to incorporate in subsequent analyses. All final variables were evaluated for correlation using a variance inflation factor (VIF) analysis (Hair et al., 1995). Once variable independence was determined, we combined all variables in a multivariate model after log transforming the dependent variable due to significant right skewness. We dredged the resulting model results using the *MuMIn* package in R to remove weakly correlated variables (Bartoń, 2012). The adjusted R^2^ was calculated for the best model to estimate the proportion of explained variance. We conducted this two‐step process because incorporating all variables into one model proved computationally difficult.

To determine the environmental drivers that predict home‐range variation locally, we subset the data into the four sites and conducted separate GLM's with the variables from the full model for each site. These models were also dredged to determine the most parsimonious models. Sex was included as a variable in the Kunene and Etosha models, where data were available. The single male from Zambezi was removed from the site analysis models, representing a limitation of our dataset. Tracking period in days was initially included as a covariate in the GLM's but was removed as it showed no effects in the models.

We exported the probability mass function (PMF) calculated from each resulting AKDE home range to provide per pixel percentages of use in different habitats. We summed the PMF within protected areas boundaries to differentiate the percentage of space use between protected and nonprotected areas, and with a particular emphasis towards evaluating the percentage of space use within national parks. All analyses were completed using the R environment for statistical computing (Version 4.0.2; R Development Core Team, 2020).

## 3 RESULTS

### 3.1 Variation in home range size

**FIGURE 2:**
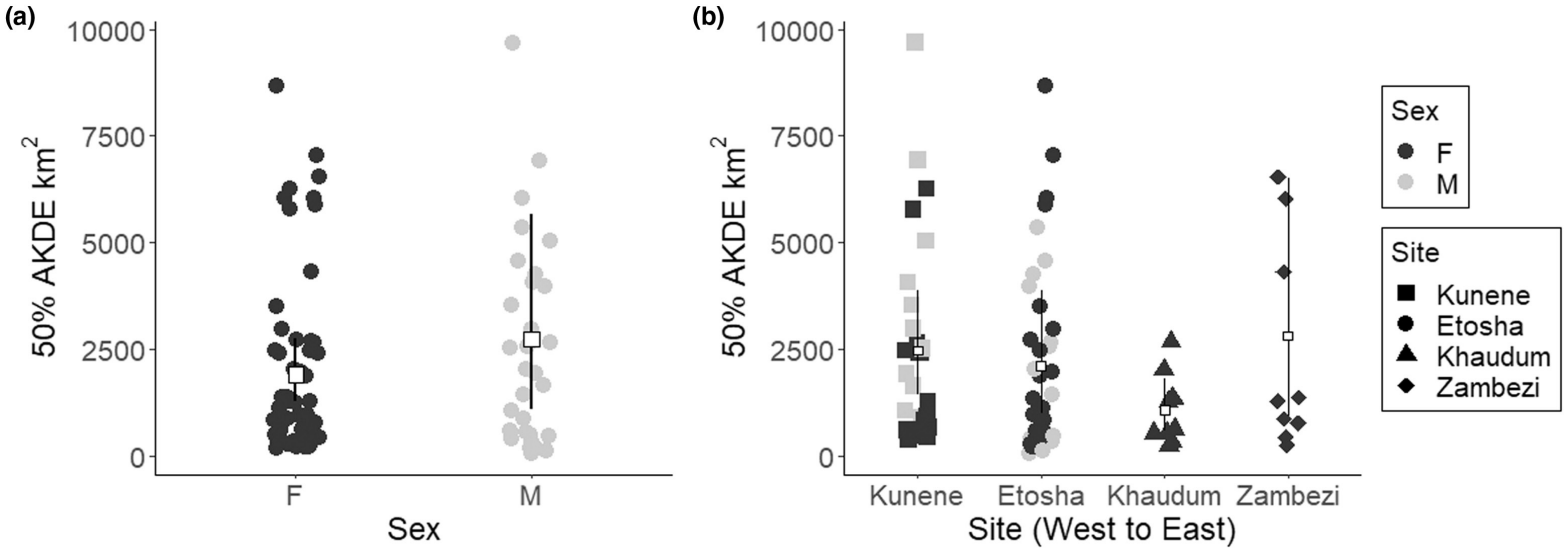
Distributions of 50% AKDE home range estimates with means for sex (a) and site (b). Sex and site indicated by color and shape (respectively) in plot B, where open squares represent mean values per site and black line the 95% CI around this mean.

The mean 50% AKDE home range estimate was 2200 km^2^ (95% CI: 1500–3100 km^2^) for all elephants included in the study. Mean and variance in home range size was greatest for males, although differences observed between sexes were nonsignificant (*F* = 1.3, *p* = .26). Male home ranges averaged 2700 km^2^ (95% CI: 1800–5700 km^2^), while female home ranges averaged 1900 km^2^ (95% CI: 1300–2700 km^2^; Figure 2a). Males had both the smallest (41 km^2^ in Etosha) and largest (9700 km^2^ in Kunene) home ranges observed. When examined by site, Zambezi elephants (1 male, 9 females) had the largest mean and variance in home range (2800 km^2^; 95% CI: 930–6500 km^2^) (Figure 2b). Conversely, Khaudum elephants (10 females) had the smallest home range areas (1100 km^2^; 95% CI: 570–1800 km^2^), with little variation amongst individuals. Confidence intervals overlapped across Kunene (12 male and 15 female) and Etosha (17 males and 22 females), with mean home range size slightly higher across Kunene (2500 km^2^; 95% CI: 1400–3900 km^2^) when compared with Etosha elephants (2100 km^2^; 95% CI: 990–3900 km^2^). Several females within Etosha had very large home ranges (>5000 km^2^), comparable with the largest home ranges in Kunene. No significant difference (*F* = 1.37, *p =* .26) in home range size existed between sites.

Minimum convex polygon (MCP) estimates were also calculated for each individual to the 95% level to allow comparison with historic studies in Namibia. The mean estimate in this study for all individuals was 3800 km^2^ (95% CI: 3200–4400 km^2^). For males, the mean MCP was 4496 km^2^ (95% CI: 3400–5600 km^2^). For females, the mean MCP was 3390 km^2^ (95% CI: 2700–4100 km^2^). Zambezi elephants had a mean MCP estimate of 5000 km^2^ (95% CI: 2400–7600 km^2^). In Khaudum, the average MCP estimate was 2200 km^2^ (95% CI: 1500–2900 km^2^). The mean 95% MCP for Etosha was 3515 km^2^ (95% CI: 2600–4500 km^2^). In Kunene, the mean MCP estimate was 4282 km^2^ (95% CI: 3300–5200 km^2^). While we focus on core home ranges from 50% AKDE estimates in this study, the difference between 95% AKDE and MCP estimates was significant (*t*_85_ = 6.9, *p* = 1.0E−9).

### 3.2 Environmental predictors for regional variation

**FIGURE 3:**
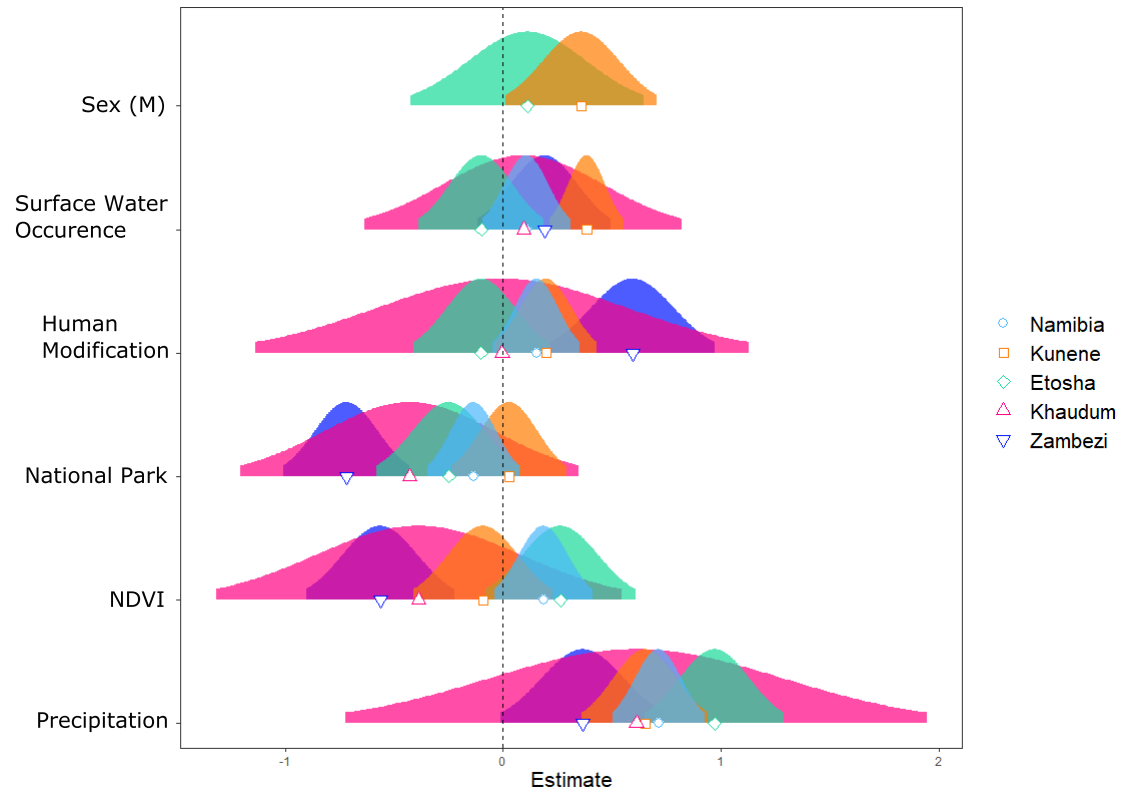
Scaled coefficients from all variable models. The shapes represent the coefficient value for each site and the curves are the theoretical normal distributions based on the 95% confidence interval of each coefficient.

Of the five variables included in the full model, precipitation had the greatest effect on home range size (*β* = 0.71, SE = 0.11). The variation of annual precipitation demonstrated a strong positive correlation with home range size, meaning years with high rainfall variability were correlated with larger elephant home ranges (Figure 3). Neither variation in vegetation productivity (*β* = 0.18, SE = 0.11), the occurrence of surface water (*β* = 0.11, SE = 0.10), nor human modification had a significant effect with home range size (*β* = 0.15, SE = 0.10; Figure 3). National parks demonstrated the greatest correlation with home range size out of all the protected area designations (e.g., communal conservancies) and concessions. Lastly, there was a negative relationship between percentage of home range in a national park and home range size, but these results were nonsignificant (*β* = −0.14, SE = 0.11; Figure 3).

Our most parsimonious model included only precipitation and vegetation (Table 3). These two variables explained approximately 53% of the regional variation in home range size. Home range size increased significantly with both variability in rainfall (*β* = 0.76, SE = 0.09) and vegetation (*β* = 0.28, SE = 0.09), though precipitation contributed more heavily to the trend.

### 3.3 Environmental predictors for site variation

The variables that best explained home range size site variation differed between study sites. The most parsimonious model for Kunene included precipitation, surface water, human impact, and sex. This model explained approximately 82% of the variation in home range size across the site (Table 3). The variables with the greatest effects were precipitation (*β* = 0.60, SE = 0.10) and surface water variability (*β* = 0.38, SE = 0.08). Greater variation in human modification was positively correlated with home range size (*β* = 0.20, SE = 0.10), as was sex (*β* = 0.35, SE = 0.17; Table 3). The best model for Etosha included only precipitation, which explained approximately 74% of home range variation in home range size (Table 3). There was a strong positive correlation between precipitation and home range size (*β* = 1.17, SE = 0.11). Sex was also included in the top model, but 95% CIs on the coefficient overlapped. The best model for Khaudum included a single variable—percentage of national park. This model explained 39% of the variation in home range size across this site (*β* = −0.54, SE = 0.2; Table 3). Khaudum had the worst model fit of any site. Zambezi models were also explained by a single variable – human impact (*β* = 1.0, SE = 0.22). Across this site, this single variable explained approximately 71% of home range variation in Zambezi (Table 3).

### 3.4 Protected areas

**FIGURE 4:**
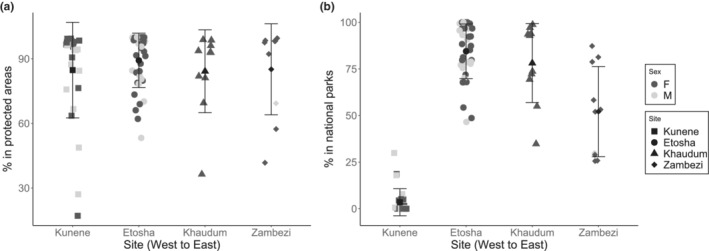
The summed Probability Mass Function (PMF) from calculated 50% home ranges within (a) protected areas and (b) national parks for each elephant. The percentage represents how much of the elephants' space use is predicted to be within different management types.

The vast majority of elephant space use was within protected area boundaries (Mean: 86%, 95% CI: 83–91%; Figure 4a). By site, the amount of space use within protected area boundaries did not differ significantly (*p* = .71). While 85% (95% CI: 76–94%) of elephant range in Kunene elephants' range were within protected areas (primarily communal conservancies), only 3.5% was within national parks (either Skeleton Coast or Etosha). Etosha elephants showed the greatest amount of space use within protected area boundaries (89%, 95% CI: 85–93%), with 84.5% (95% CI: 80–89%) of space use within Etosha National Park. In Khaudum, 84% (95% CI: 71–98%) of elephant space use was within protected area boundaries, skewed by a single individual who spent a large amount of time dispersing into unprotected lands. In Zambezi, 85% (95% CI: 70–100%) of elephant space use was within protected areas, 52.0% (95% CI: 35–69%) of which was in national parks (Figure 4).

## 4 DISCUSSION

Elephant home ranges in northern Namibia varied widely across local and regional scales. Our results highlight, however, that much of this variation can be explained by a few key environmental variables. On a regional scale, we found that precipitation and vegetation explained 53% of the variation in home range size. At the site level, home range differences were also influenced by the interaction between rainfall and human development. For example, in the wetter and more populous Zambezi, human modification strongly predicted home range size, while precipitation did not. Conversely, precipitation strongly affected elephant home ranges in the drier and less populated sites of Kunene and Etosha. Elephants in all study areas maintained high fidelity to protected areas, especially national parks, which in part is due to fencing in Etosha and Khaudum. These results suggest that elephant space use in Namibia is primarily driven by natural dynamics (e.g., precipitation), though human modification is impacting some sites. Examining environmental variables on multiple scales provides a method to investigate how widely humans are impacting elephant space use compared with other environmental variables, and to identify sites where further work is needed to mitigate negative impacts from humans. Furthermore, Namibia's natural gradient from xeric to more mesic habitats allowed us to ascertain how these factors interact differently in geographically close, but distinct locations to influence home range, which may have implications for conservation.

Our MCP estimates were smaller on average in Kunene and Etosha than previously published range sizes (Lindeque & Lindeque, 1991; Leggett, 2006a, 2006b; Table 2), but similar in that our estimates varied widely between individuals from the same site (i.e., Etosha: 240–13,000 km^2^). Our 95% AKDE estimates were more than double MCP estimates for the same individuals (Table 2). This is consistent with studies which have compared AKDE to traditional metrics (Moßbrucker et al., 2016; Noonan et al., 2020). Our AKDE and MCP estimates were also larger than local convex hull estimates for the same populations from Roever et al. (2012), which is consistent with comparison of these methods in Noonan et al. (2019).

**TABLE 2:** Elephant home range estimates from Kunene, Etosha, Khaudum and Zambezi using autocorrelated kernel density estimation (AKDE), fixed kernel density estimates (KDE), ellipse, or minimum convex polygon (MCP) from this study and previous studies

Location	No./sex	Home range (km^2^)	HR Mean	Method	Citation
Kunene	4 M	2300–9600 (3000–15,000)	5000 (6600)	95% MCP (95% Ellipse)	Lindeque and Lindeque (1991)
Kunene	2 F, 6 M	870–13,000	3100	95% MCP/KDE	Leggett (2006a, 2006b)
**Kunene**	**15 F, 12 M**	**370–9700 (1500–37,000) 1600–11,000**	**2500 (11,000) 4300**	**50% AKDE (95% AKDE) 95% MCP**	**This study**
Zambezi	8 F, 1 M	580–5600	2600	100% MCP	Rodwell (1995)
Zambezi	1 F, 2 M	5000–20,000 (1600–2000)	13,000 (1700)	95% MCP (Grid)	von Gerhardt‐Weber (2011)
Zambezi and Khaudum			1431	Local convex hull	Roever et al. (2012)
**Zambezi**	**9 F, 1 M**	**250–6500 (1400–25,000) 1200–13,000**	**2800 (11,000) 5000**	**50% AKDE (95% AKDE) 95% MCP**	**This study**
Etosha, Khaudum, Ngamiland	3 F per site	~1000–2500	~1800	95% KDE	Young et al. (2009)
Etosha and Kunene			573	Local convex hull	Roever et al. (2012)
Etosha	3 M	2100–11,000 (2900–19,000)	6971 (11,000)	95% MCP (95% Ellipse)	Lindeque and Lindeque (1991)
**Etosha**	**22 F, 17 M**	**41–8700 (280–38,000) 240–13,000**	**2000 (9200) 3500**	**50% AKDE (95% AKDE) 95% MCP**	**This study**
**Khaudum**	**10 F**	**240–2700 (3000–12,000) 1200–4200**	**1100 (4400) 2200**	**50% AKDE (95% AKDE) 95% MCP**	**This study**

*Note*: Bold text is highlighting which rows reflect data form this study.

**TABLE 3:** Best GLM models for each site with coefficient values and adjusted *R* ^2^

Site	Variables	Coefficients	Adjusted *R* ^2^
Full Model	SD Annual precipitation	0.76***	0.53
	SD NDVI	0.28**	
Kunene	SD Annual precipitation	0.60***	0.82
	SD Occurrence of water	0.38***	
	SD Human modification	0.20*	
	Sex	0.35	
Etosha	SD Annual precipitation	1.17***	0.74
Khaudum	National parks %	−0.54**	0.39
Zambezi	SD human modification	1.00**	0.71

*Note*: *p*‐values indicated by <.001***, .01**, and .05*.

Despite high individual variation, regional differences in home range size were clearly correlated with precipitation and NDVI, which we anticipated given Namibia's pronounced rainfall gradient (Appendix S2: Figure A1). This finding is consistent with other studies of megaherbivores in Africa (e.g., Knüsel et al., 2019), in which mean annual rainfall explained 74% of the variation in giraffe (*Giraffa camelopardalis*) home range size. Previous studies of elephants have linked precipitation to elephant movements and space use. Young and Van Aarde (2010), for example, found that the daily displacement distance of elephants decreased with increased rainfall across 13 southern African study sites (including Etosha), while Grogan et al. (2020) found that annual precipitation was the only variable found to be negatively correlated with annual home range size.

While we did not hypothesize that vegetation would be important regionally, the inclusion of NDVI in the best regional model is not surprising. High‐quality vegetation is known to be an important grazing resource for herbivores, which impacts their space use (McLoughlin & Ferguson, 2000; Tufto et al., 1996; van Beest et al., 2011). Across our study sites, vegetation productivity follows a similar West–East gradient in relation to the precipitation gradient in Namibia. Elephants are known to be particularly adept at seeking out highly productive patches of vegetation throughout the year (Loarie et al., 2009). Other studies, however, have found that elephant movements cannot be solely attributed to vegetation productivity, with individuals having complex foraging strategies which are not uniform in space or time (Boettiger et al., 2011). This may explain why elephants prefer areas with higher landscape heterogeneity (de Beer & van Aarde, 2008) and why standard deviation, and not mean, of NDVI outperformed other metrics for capturing the relationship between space use and vegetation productivity.

In a recent continental scale analysis, Wall et al. (2021) found that human footprint was the dominant factor correlated with annual home range size, while other factors like water availability and vegetation productivity, showed strong correlation with home range size on short temporal scales. Conversely, human impacts did not have a significant effect on home range size at a regional level in our model. This, in part, could be due to two of our four study areas being within national park boundaries where human access/disturbance is limited. Additionally, Wall et al. (2021) spans a wider gradient of human impacts than our study and included sites where there is higher human footprint along park boundaries. While human impacts do vary across our study area, Namibia has the lowest human population density of any African country and one of the lowest human population densities in the world (3 people per km^2^; United Nations Populations Division, 2022).

Human modification index was included in the top model of the Zambezi and Kunene sites, both of which have a mosaic of protected and unprotected areas. Kunene is the most arid site with relatively low human population density. Although human modification was included in the top model, this parameter was secondary to ecological factors. In contrast, the Zambezi site has the highest human population density (6.2 people/km^2^; Namibia Statistics Agency, 2011) and demonstrated the strongest relationship between elephant range size and human modification.

In Kunene, precipitation and surface water had a greater effect on home range than human impact, indicating ecological factors were more critical to structuring range use. The scarcity of water on the landscape may contribute to the inclusion of human impacts in the model because human‐wildlife interactions around limited water sources can result in conflict. In an attempt to mitigate and reduce the intensity of localized human‐elephant conflict in the Kunene, some elephants in two affected areas were captured and sold in 2021. Despite benefits, the cost of sharing a landscape with elephants can be high, with relatively few directly benefiting from revenues generated (Schnegg & Kiaka, 2018). The cost/benefit ratios are highly variable between conservancies with some experiencing large profit margins while others suffer disproportionate losses from human‐wildlife conflict (Brown, 2011). While Kunene elephant numbers have increased overall in the past decades (Schnegg & Kiaka, 2018), evidence exists that elephants in some Kunene conservancies experience higher levels of stress and potentially lower calf recruitment compared with those in Etosha (Hunninck et al., 2017). Notable declines of elephants in the Hoarusib, Hoanib, and Uniab river systems have occurred (Ramey & Brown, 2019).

Anthropogenic disturbances have caused significant changes in vegetation structure and composition in Kunene, which will only further degrade the landscape without intervention (Inman et al., 2020). Degradation and restriction of movement could further endanger this population. Kunene elephants only spent 3.5% of their time in national parks (Etosha and/or Skeleton Coast). The capacity for either national park to function as refugia is limited because Etosha is fenced and Skeleton Coast is extremely arid. Etosha could function as a refugium if a functional corridor were established between western Etosha and the Hobatere concession.

Human impacts were the only covariate in the top model of home range variation in Zambezi. Greater variance in human modification is positively associated with home range size and may indicate elephants are moving through areas of high human modification to access fragmented patches of habitat between human settlements. Similar results were found for giraffes (*Giraffa camelopardalis*) whose home range sizes were negatively correlated with distance to densely populated towns (Knüsel et al., 2019). Our findings also support previous studies which specify Zambezi as an area of high human‐wildlife conflict with restrictions on animal movement (Stoldt et al., 2020). Despite high human modification, relatively high elephant numbers are sustained. Occupancy of this area by elephants and other large mammals has increased in recent decades but is more heavily constrained and fragmented by agricultural expansion and fences (Stoldt et al., 2020). Existing corridors should be carefully monitored, maintained and protected to preserve connectivity in the face of human pressures (Brennan et al., 2020). This is especially key because Zambezi sits at the heart of the KAZA Transfrontier Conservation Area and connects habitat between Angola, Zambia, and Botswana.

Despite indicating human impacts on elephant space use, our results highlight the importance of protected areas, especially national parks, for elephants. There was especially high fidelity to Etosha and Khaudum by elephants, which points to the success of these parks in meeting elephants' needs. Vegetation productivity and persistence are higher in national parks compared with buffer areas throughout Southern Africa (Herrero et al., 2016). The persistence of vegetation, related to restricted human use, may in part explain why elephants remain within national parks as elephants are known to respond strongly to long‐term patterns in productivity (Tsalyuk et al., 2019). Additionally, national parks like Etosha and Khaudum have well‐maintained artificial waterholes, which are known to decrease home range size by decreasing the distance that animals must travel to access water resources (de Beer & van Aarde, 2008). Etosha has 60 artificial boreholes, contact springs as well as artesian water, which provide water all year. Khaudum has 11 artificial water sources. Water provisioning increases elephant densities locally and changes their distribution on the landscape, though this impact is mitigated by other factors such as forage quality (Chamaillé‐Jammes et al., 2007; Smit et al., 2007). In Etosha, elephants prefer areas within 4 km of water throughout the year (de Beer et al., 2006), but attraction to these points may be offset by degradation of nearby vegetation from heavy foraging (Shannon et al., 2009).

While more spatially dense resources may contribute to smaller home ranges, elephants in unfenced protected areas are known to disperse, using unprotected lands as corridors while maintaining core areas within parks (Douglas‐Hamilton et al., 2005). Etosha is completely fenced, while Khaudum is fenced along its eastern border with Botswana, which likely contributes to high fidelity to the parks. The high fidelity of the Khaudum elephants contrasts to Buchholtz et al. (2019), which found frequent movement between Khaudum, northern Botswana, and Zambezi. This difference could be indicative of sex‐specific movement patterns as our sample only included females. Elephants in Etosha showed little dispersal with only a few venturing outside park boundaries. Several Khaudum elephants, however, dispersed south into neighboring Nyae Nyae conservancy and one individual even spent considerable time in unprotected lands. Fencing in Etosha may restrict dispersal, especially in the wet season when elephants are known to “bunch‐up” against fences (Loarie et al., 2009). But, because there is no significant difference in home range size between regions, it is unlikely that fencing is completely restrictive and causing uncommonly small home ranges. Better measures of protection in national parks, in part a result of maintained fences, may be an important factor explaining high fidelity.

In conclusion, our results demonstrate the variation in drivers of elephant range size across ecological and human modification gradients. In arid sites, which tended to have larger home ranges and lower human density, human activity became more influential to recorded range sizes. In the highest human density area, human activity was the sole correlate of elephant range size in our top model. Interestingly, home range estimates of elephants have not altered drastically from estimates 30 years ago. A key concern going forward is the interaction and competition for space between growing human and elephant populations. Our results highlight the critical role government‐ and community‐run protected areas play in the current Namibian elephant distribution. Maintaining healthy populations of this wide‐ranging megaherbivore is no easy feat, but Namibia's success should be acknowledged in the face of continent‐wide declines of this endangered species.

## AUTHOR CONTRIBUTIONS

**Lorena Benitez:** Data curation (equal); formal analysis (lead); methodology (equal); writing – original draft (lead); writing – review and editing (equal). **J. Werner Kilian:** Conceptualization (equal); data curation (equal); funding acquisition (equal); investigation (lead); methodology (equal); project administration (equal); supervision (equal); writing – original draft (supporting); writing – review and editing (equal). **George Wittemyer:** Conceptualization (equal); formal analysis (equal); methodology (equal); supervision (equal); writing – original draft (supporting); writing – review and editing (equal). **Lacey F. Hughey:** Conceptualization (supporting); formal analysis (supporting); methodology (supporting); supervision (supporting); validation (supporting); visualization (supporting); writing – original draft (supporting); writing – review and editing (equal). **Christen H. Fleming:** Data curation (supporting); formal analysis (supporting); methodology (supporting); software (equal); writing – original draft (supporting); writing – review and editing (equal). **Peter Leimgruber:** Formal analysis (supporting); investigation (supporting); writing – review and editing (equal). **Pierre du Preez:** Investigation (equal); methodology (equal); resources (equal). **Jared A. Stabach:** Conceptualization (equal); data curation (equal); formal analysis (equal); funding acquisition (equal); investigation (equal); methodology (equal); project administration (equal); resources (equal); supervision (lead); validation (equal); visualization (equal); writing – original draft (supporting); writing – review and editing (equal).

## CONFLICT OF INTEREST

The authors declare that there is no conflict of interest.

## Supporting information


Appendix S1

Appendix S2

## Data Availability

In view of poaching concerns, the datasets generated or analyzed during the current study are not publicly available but can be made available upon reasonable request via Movebank (ID: 1807299477).
