# Supplementary material for: Precipitation, vegetation productivity, and human impacts control home range size of elephants in dryland systems in northern Namibia
Source: Ecol Evol. 2022 Sep 13;12(9):e9288. doi: 10.1002/ece3.9288 (PMC9471278; doi:10.1002/ece3.9288)
Supplement: Supplementary file 2 — Appendix S2 [file ECE3-12-e9288-s002.pdf]

Appendix A

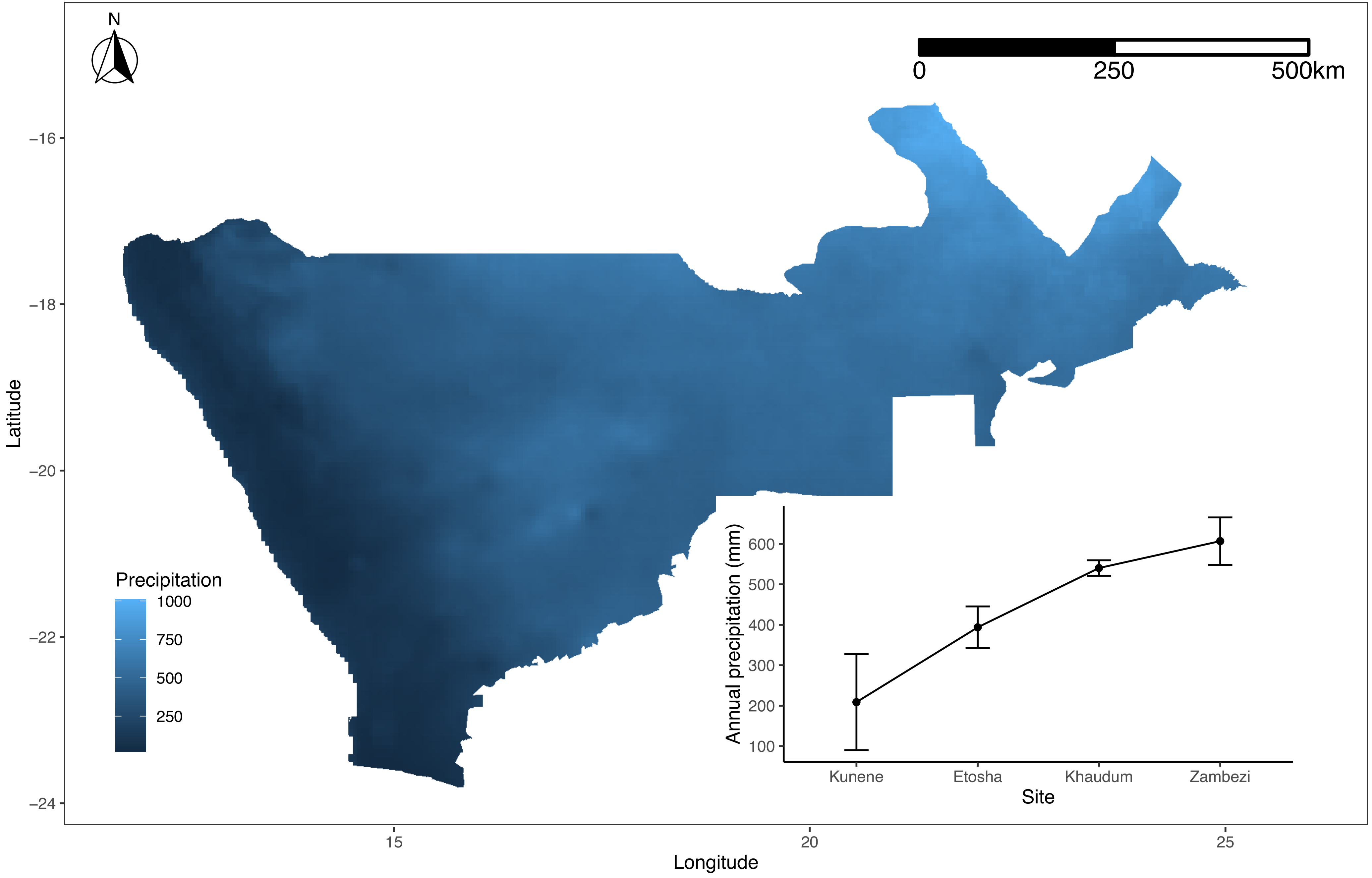

Figure A1: Annual precipitation (mm) for the study region calculated from CHIRPS (Funk et al., 2015). The graph represents a summary of the values in each of the four sites. The error bars represent one standard deviation above and below the mean. The average and standard deviations were calculated using the minimum convex polygon (MCP) of all GPS points from that site inclusive of a five-kilometer buffer.

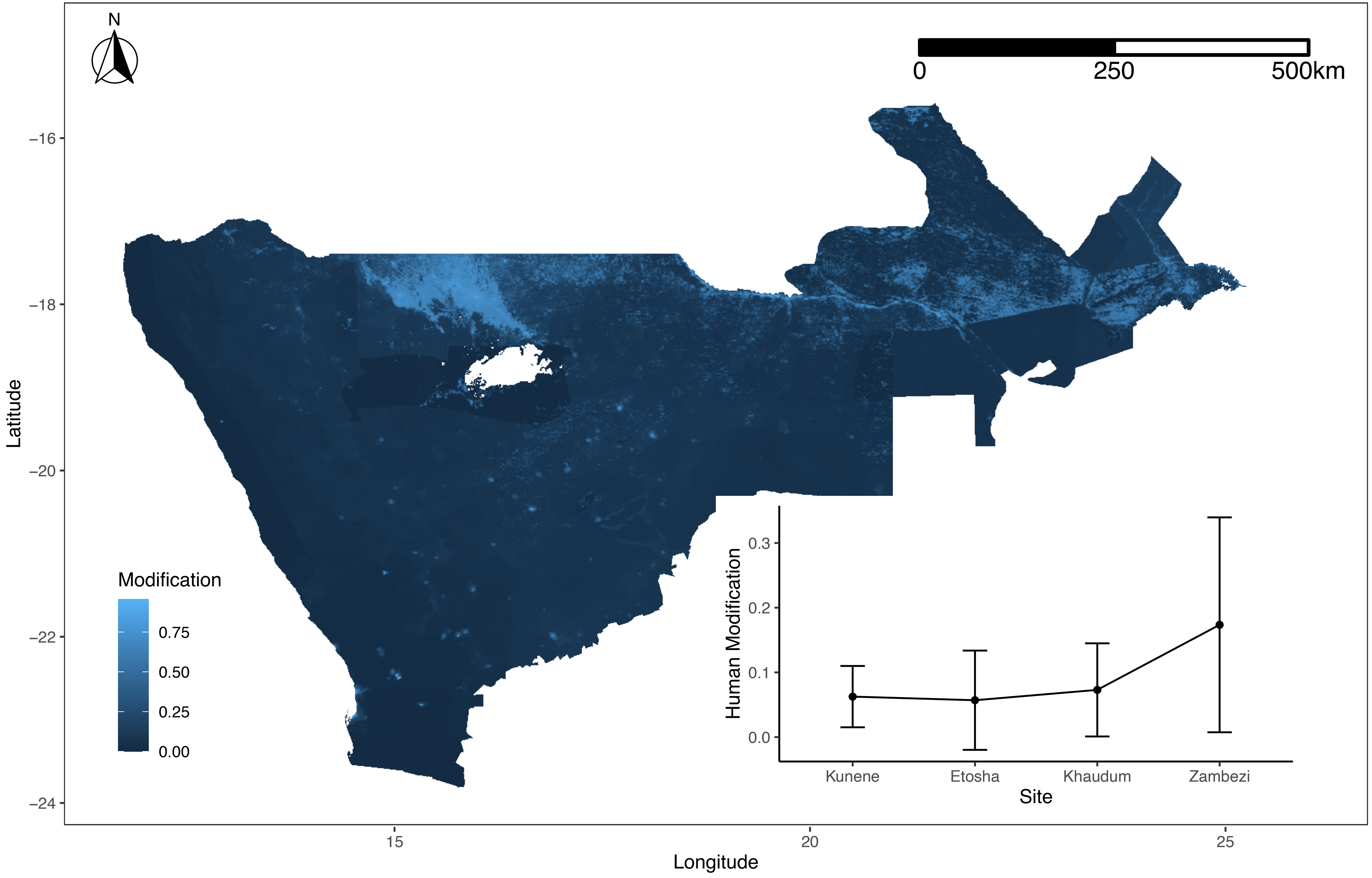

Figure A2: Human modification for the study region from the Human Modificiation Index (Kennedy et al., 2019). The graph represents a summary of the values in each of the four sites. The error bars represent one standard deviation above and below the mean. The average and standard deviations were calculated using the minimum convex polygon (MCP) of all GPS points from that site inclusive of a five-kilometer buffer.

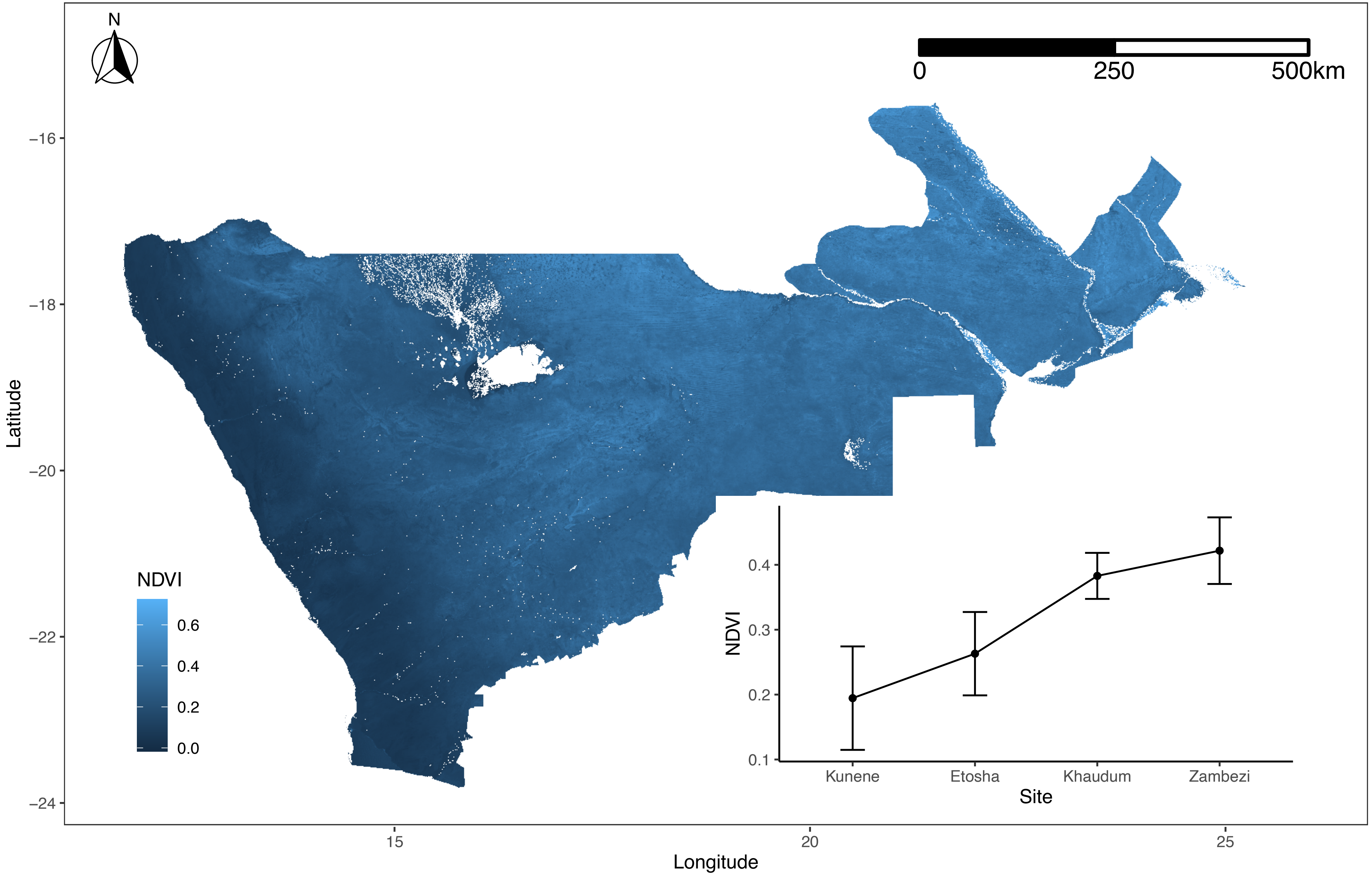

Figure A3: NDVI for the study region with pixels that represent surface water removed. The graph represents a summary of the values in each of the four sites. The error bars represent one standard deviation above and below the mean. The average and standard deviations were calculated using the minimum convex polygon (MCP) of all GPS points from that site inclusive of a five-kilometer buffer.

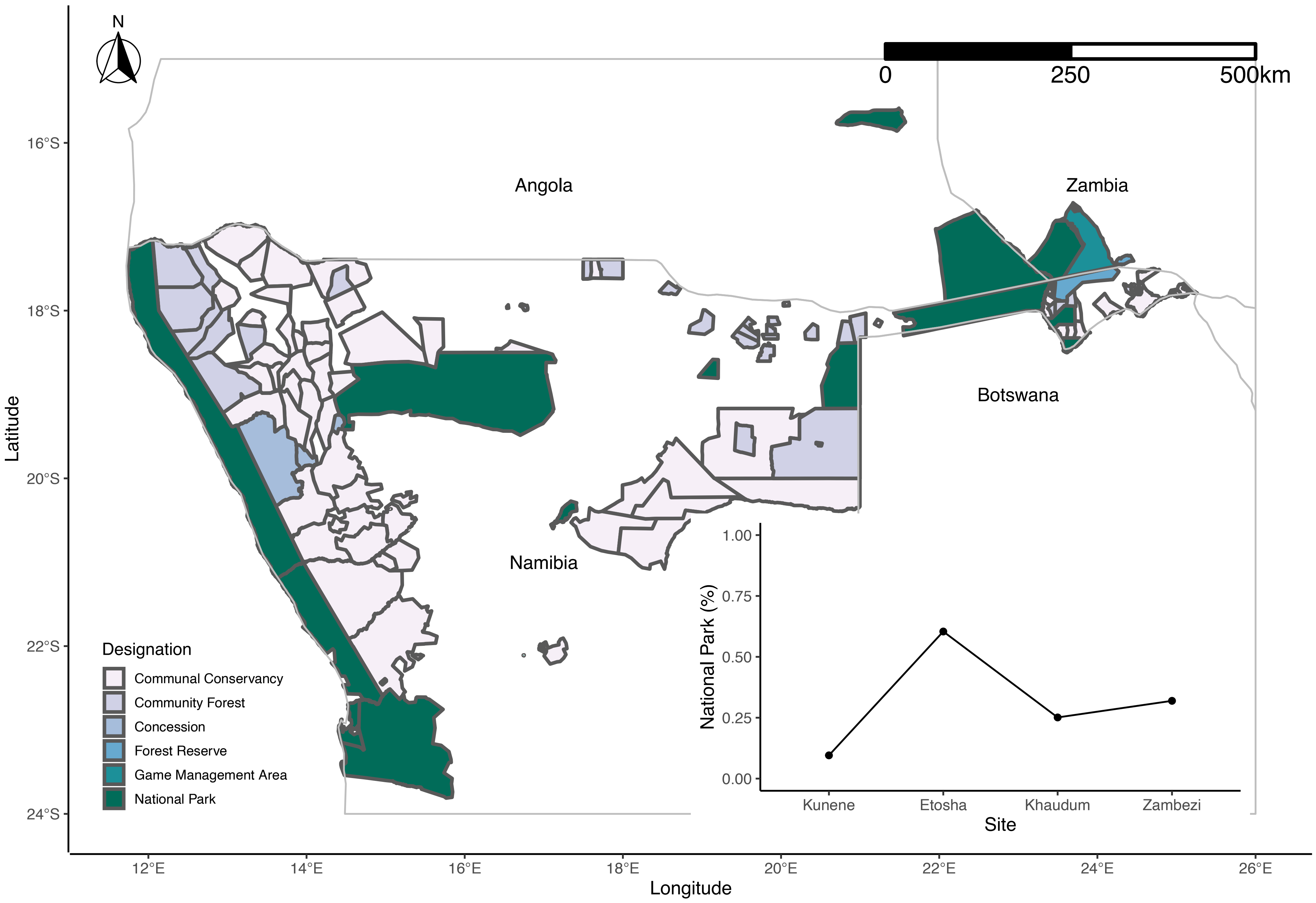

Figure A4: Protected areas with the study region with nationals parks in green and communal conservancies in pink. The graph represents a summary of the values in each of the four sites. The error bars represent one standard deviation above and below the mean. The average and standard deviations were calculated using the minimum convex polygon (MCP) of all GPS points from that site inclusive of a five-kilometer buffer.

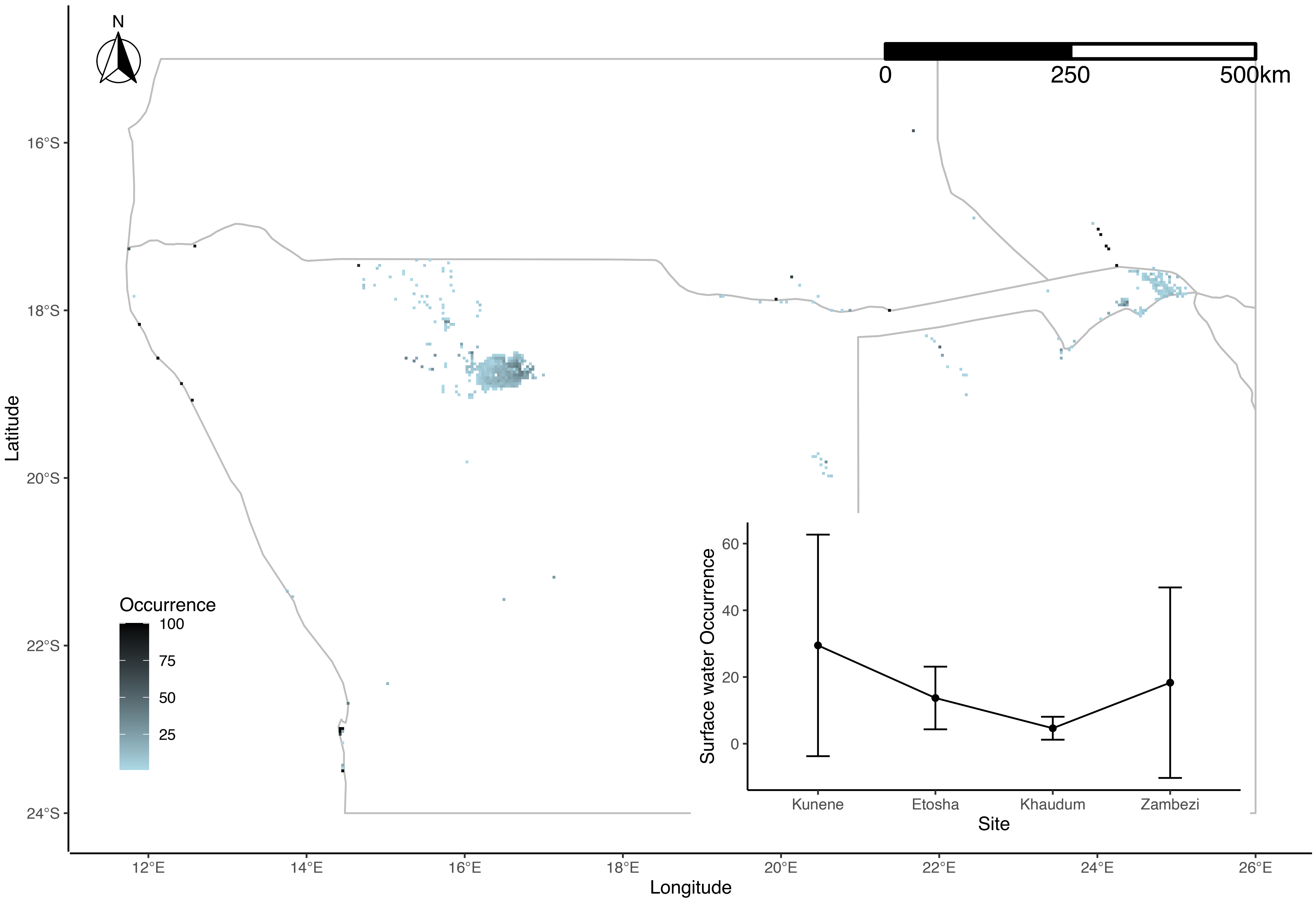

Figure A5: Surface area for the study region calculated from the JRC Global Surface Water Mapping Layers (Pekel et al., 2016). The graph represents a summary of the values in each of the four sites. The error bars represent one standard deviation above and below the mean. The average and standard deviations were calculated using the minimum convex polygon (MCP) of allGPS points from that site inclusive of a five-kilometer buffer.
